# Nuclear Pore Complexes Cluster in Dysmorphic Nuclei of Normal and Progeria Cells during Replicative Senescence

**DOI:** 10.3390/cells10010153

**Published:** 2021-01-14

**Authors:** Jennifer M. Röhrl, Rouven Arnold, Karima Djabali

**Affiliations:** Epigenetics of Aging, Department of Dermatology and Allergy, TUM School of Medicine, Technical University of Munich (TUM), 85748 Garching, Germany; jennifer.roehrl@tum.de (J.M.R.); rouven.arnold@tum.de (R.A.)

**Keywords:** progerin, nuclear pore, mitosis, replicative senescence, progeria, nuclear envelope, nucleus

## Abstract

Hutchinson-Gilford progeria syndrome (HGPS) is a rare premature aging disease caused by a mutation in *LMNA*. A G608G mutation in exon 11 of *LMNA* is responsible for most HGPS cases, generating a truncated protein called “progerin”. Progerin is permanently farnesylated and accumulates in HGPS cells, causing multiple cellular defects such as nuclear dysmorphism, a thickened lamina, loss of heterochromatin, premature senescence, and clustering of Nuclear Pore Complexes (NPC). To identify the mechanism of NPC clustering in HGPS cells, we evaluated post-mitotic NPC assembly in control and HGPS cells and found no defects. Next, we examined the occurrence of NPC clustering in control and HGPS cells during replicative senescence. We reported that NPC clustering occurs solely in the dysmorphic nuclei of control and HGPS cells. Hence, NPC clustering occurred at a higher frequency in HGPS cells compared to control cells at early passages; however, in late cultures with similar senescence index, NPCs clustering occurred at a similar rate in both control and HGPS. Our results show that progerin does not disrupt post-mitotic reassembly of NPCs. However, NPCs frequently cluster in dysmorphic nuclei with a high progerin content. Additionally, nuclear envelope defects that arise during replicative senescence cause NPC clustering in senescent cells with dysmorphic nuclei.

## 1. Introduction

Hutchinson-Gilford progeria (HGPS) is a rare premature aging disease, caused by a de novo point mutation in the lamin A gene *LMNA* G608G (GGC → GGT) [1,2]. The mutation introduces a cryptic splice site, which results in the deletion of 50 amino acids in the carboxy-terminus of pre-Lamin A (preLA) [1]. This deletion removes the recognition site of the protease ZMPSTE24, thereby creating a permanently farnesylated preLA mutant, progerin, which remains attached to the nuclear envelope (NE) [3,4]. Progerin causes various defects in cells, including an abnormal nuclear shape [5,6], a thickened nuclear lamina, loss of peripheral heterochromatin, and clustering of several proteins [7,8].

One affected protein complex in HGPS cells is the nuclear pore complex (NPC) [5,7], which functions as a link between the cytosol and nucleoplasm, allowing free diffusion of components approximately 5 nm in diameter or ≤60 kDa as well as active transport via nuclear transport receptors for larger molecules [9].

**Figure 1:**
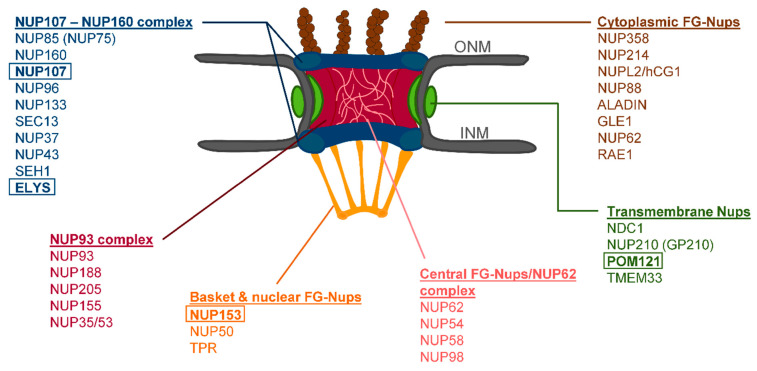
Schematic representation of the nuclear pore complex (NPC) architecture. The cytoplasmic and nucleoplasmic ring (NUP107-160 complex) are shown in blue, the transmembrane nucleoporins (NUPs) in green, the central ring (NUP93 complex) in red, the cytoplasmic filaments in brown, the nuclear basket in orange, and the central FG NUPs in pink. Boxes indicate NUPs analyzed in this study.

The NPC is a large complex of approximately 112 MDa [10] containing around 30 subunits (Figure 1), called nucleoporins (NUP). It presents an eightfold rotational symmetry [9,11], and the structure can be divided into substructures: the inner pore ring (NUP93 complex, NUP62 complex), nuclear and cytoplasmic rings (NUP107-160-complex), nuclear basket and cytoplasmic filaments [12]. It is anchored to the NE via the transmembrane NUPs, NDC1, POM121, and GP210 [13].

The NPC is assembled at two different stages of the cell cycle: de novo assembly during interphase and reassembly following open mitosis [14]. Post-mitotic assembly is a highly ordered process, in which different subcomplexes and NUPs are recruited sequentially [14]. The current theory of post-mitotic assembly is that NPCs are preformed on the surface of chromatin and subsequently enclosed by the reformation of the NE at the end of mitosis [14].

ELYS, a member of the NUP107-160 complex containing an AT-hook DNA-binding domain [15], is the first NUP seeded on anaphase chromosomes [16]. After binding to DNA, ELYS recruits the remainder of the NUP107-160 complex (Figure 1) [16,17]. Next, two members of the nuclear basket, NUP153 and NUP50, are partially recruited to the chromatin periphery [18,19,20], followed by two transmembrane NUPs, NDC1 and POM121, in early to late anaphase [16,18,21,22,23,24]. Subsequently NUP53, part of the NUP93 complex (central channel, Figure 1), is recruited by NDC1 [25,26]. In turn this leads to the binding of NUP155 and NUP93, completing the NUP93 complex (Figure 1) [25]. Nuclear import is established by the recruitment of NUP62 complex (Figure 1) by NUP93 in the telophase [18,27]. The remaining members of the NPC, mainly the cytoplasmic filament NUPs (Figure 1) and the remainder of the nuclear basket NUPs’ (NUP153, NUP50, and TPR) are assembled in late telophase and are completed only in early G1 [18].

Previously, we reported that progerin interferes with NE reassembly following mitosis, and one of the most affected proteins is SUN1 [8]. SUN1 acts in concert with a transmembrane NUP, POM121, in interphase NPC assembly [28,29]. Furthermore, it has been reported that SUN1 preferentially interacts with preLA [30,31]. PreLA only transiently exists in normal cells, raising the question of whether SUN1 targets preLA to the inner nuclear envelope (INM) and serves as a nucleation site for A-type lamin assembly. In HGPS cells, progerin tightly bound to SUN1 may indirectly trap nearby NPCs by reducing SUN1 mobility [31]. If these progerin-SUN1-NPC interactions occur during NE reformation in mitosis, this may result in NPC clusters [5].

In this study, we focused on identifying the mechanism of nuclear pore clustering in HGPS cells. Using unsynchronized primary fibroblast cultures, we examined NPC reformation during mitosis in control and HGPS nuclei with immunocytochemistry. To identify possible spatiotemporal alterations in the NPC assembly in mitotic HGPS cells caused by progerin, we tracked different NPC subunits belonging to the NUP107-160 complex, the nuclear basket, and one transmembrane NUP relative to progerin and other nuclear components. Next, we tracked NPCs distribution in both normal and HGPS cells during interphase and senescence.

## 2. Materials and Methods

### 2.1. Cell Culture

The HGPS fibroblast cell lines HGADFN003, HGADFN127, HGADFN178, and HGADFN188, carrying the *LMNA* mutation G608G, were acquired from The Progeria Research Foundation Cell and Tissue Bank (https://www.progeriaresearch.org/). Control fibroblasts GMO1651C and GMO1652C were acquired from the Coriell Institute for Medical Research (Camden, NJ, USA). Cells were cultured in DMEM containing 15% fetal bovine serum (FBS), 1% glutamine, 1% penicillin, and 0.5% streptomycin (growth medium). Cells for immunofluorescence of mitotic cells were seeded on glass coverslips at a density of 3000 cells/cm^2^ and fixed after 48 h. To increase the number of mitotic cells for some experiments, cells were synchronized by serum starvation. For this, cells were seeded at a density of 4000 cells/cm^2^ and cultured in growth medium for 24 h, followed by incubation in starvation medium, containing 0.1% FBS, for 72 h. Thereafter, cells were released by incubation in normal growth medium and fixed after 28 to 31 h of cell release. Cells used for statistical analysis were seeded at a density of 2000 cells/cm^2^ in normal medium and fixed on day 4.

### 2.2. Senescence Associated β-Galactosidase Assay

The Senescence Detection Kit I (PromoKine, PK-CA577-K320) was used according to the manufacturer’s instructions. Blue cells were manually counted, with at least 300 cells counted per experiment in triplicate. To compare progerin levels in β-galactosidase positive cells, HGPS cells were first stained using the Senescence Detection Kit and then permeabilized and fixed with ice-cold methanol (MeOH) for 10 min at −20 °C. After permeabilization with MeOH, cells were blocked and labeled according to the immunocytochemistry protocol in Section 2.3.

### 2.3. Immunocytochemistry

Cells grown on glass coverslips were fixed in ice-cold MeOH or 4% paraformaldehyde (PFA). Cells were either fixed at −20 °C for 10 min (MeOH) or at room temperature (RT) for 15 min (PFA) and rinsed with PBS. Following PFA fixation, cells were permeabilized with 0.2% Triton X-100 in PBS for 10 min at RT and rinsed with PBS.

The primary antibodies used in this study were: mouse-anti-NPC (BioLegend, San Diego, CA, USA, MMS-120P, mAb414, 1:1500), rabbit-anti-POM121 (Sigma-Aldrich, Taufkirchen, Germany, SAB2700248, 1:800), mouse-anti-NUP107 (Thermo Fisher Scientific, Waltham, MA, USA, MA1-10031, 1:500), rabbit-anti-SUN1 (Sigma-Aldrich, HPA008346, 1:600), rabbit-anti-progerin S9 or S5 [32], rabbit-anti-ELYS (Bethyl Laboratories, Montgomery, TX, USA, A300-166A, 1:500), mouse-anti-Lamin A (Abcam, Cambridge, UK, 133A2, 1:500), mouse-anti-p16^INK4A^ (Sigma Aldrich, St. Louis, MO, USA, P 0968, 1:250), and mouse-anti-p21 (Santa Cruz Biotechnology, Dallas, TX, USA, sc-6246, 1:250). The secondary antibodies used were affinity-purified Alexa Fluor^®^ 555 or 488 conjugated anti-rabbit/mouse antibodies (Life Technologies, Carlsbad, CA, USA, A21206 anti-rabbit-488, A21202 anti-mouse-488, A31572 anti-rabbit-555, A31570 and anti-mouse-555, 1:800/1000).

Cells were blocked for 1 h in 10% FBS in PBS. Primary antibodies were diluted in the blocking buffer at the concentration listed above, and cells were incubated for 2 h at RT or overnight at 4 °C. Next, cells were rinsed with PBS-, before adding secondary antibodies diluted in the blocking buffer for 1 h at RT. Following incubation with the secondary antibody, cells were rinsed with PBS. Coverslips were counterstained with DAPI Vectashield mounting medium (Vector Inc., Burlingame, CA, USA, VEC-H-1200) and mounted. Images were obtained using an Axio Imager D2 fluorescence microscope (AxioCam MRm, Objective X63 oil NA 1.4, Carl Zeiss, Oberkochen, Germany) and a Leica SP8 Lightning confocal microscope (objective X63 oil NA 1.4, Leica, Wetzlar, Germany).

### 2.4. Image Analysis

Images were analyzed and brightness/contrast adjusted with Fiji [33] and imported into Adobe Photoshop CC 2017 for presentation. 3D rendering was performed with Imaris (Oxford Instruments, Zurich, Switzerland).

### 2.5. Statistical Evaluation and Graphs

To determine the relationship between NPC clustering, senescence and progerin levels, cells were analyzed as follows: 300 cells per experiment were counted per cell strain (two controls and two HGPS). Three replicates were performed adding to a total of at least ~1000 cells counted per condition. Primary fibroblast cultures were examined at an index of senescence associated β-galactosidase positive cells of either ≤5% (young cultures) or ≥30% (considered old cultures). Nuclei were manually counted with an Axio Imager D2 fluorescence microscope (AxioCam MRm, Carl Zeiss using the 40X oil objective (NA 1.3, Carl Zeiss). Representative images were obtained with the same microscope using the 63X oil objective (NA 1.4, Carl Zeiss).

All results are presented as mean ± SD and compared using the Student’s *t*-test, repeated measures one-way or two-way ANOVA, depending on the type of comparison. Symbols used for indicating statistical significance include: ns, not significant, *p* > 0.05, * *p* ≤ 0.05, ** *p* ≤ 0.01, and *** *p* ≤ 0.001. Calculations and graphs were performed using Prism version 6.01 (GraphPad, San Diego, CA, USA).

### 2.6. SDS-PAGE and Westernblot

Cell pellets were extracted in Laemmli sample buffer (BioRad, Hercules, CA, USA), the total protein extract concentrations were determined by a Bradford assay (BioRad,), proteins were separated on 7% SDS-PAGE gels (BioRad) and transferred to a nitrocellulose membrane as described previously [34]. The blots were blocked for 1 h at RT in 5% milk in TBS Tween20 (TBS-T) and probed with the following primary antibodies for 1 h at RT or ON at 4 °C: mouse-anti-β-actin (Sigma-Aldrich, A1978-200UL, 1:5000), rabbit-anti-POM121 (Sigma-Aldrich, SAB2700248, 1:1000), rabbit-anti-Lamin A/C (Santa Cruz Biotechnology, E-1, 1:1000), and rabbit-anti-NUP153 (Bethyl, A301-788A, 1:1000). Membranes were washed with TBS-T and incubated for 1 h at RT with appropriate Peroxidase-conjugated secondary antibodies (Jackson Immuno Research, Westgrove, PA, USA). Blots were developed using enhanced chemiluminescence detection system (ECL substrate; BioRad) and ChemiDoc^TM^ MP (BioRad). Images were analyzed with Fiji [33] and signals were quantified by normalizing to β-actin.

## 3. Results

### 3.1. Progerin Aggregates in the Cytoplasm of Mitotic Cells Do Not Colocalize with NUP107

Previously, we reported that progerin aggregates in the cytoplasm of mitotic HGPS cells interfere with the localization of various proteins including LA, SUN1, LB1, and emerin throughout mitosis [8]. We hypothesized that NPC clustering can be attributed to progerin affecting its post-mitotic reassembly. Since no thorough investigation of NUP distribution has been performed yet, we screened several components located in the different NPC subcompartments as indicated in the schematic representation in Figure 1, to detect possible changes in their localization during NPC reassembly.

**Figure 2:**
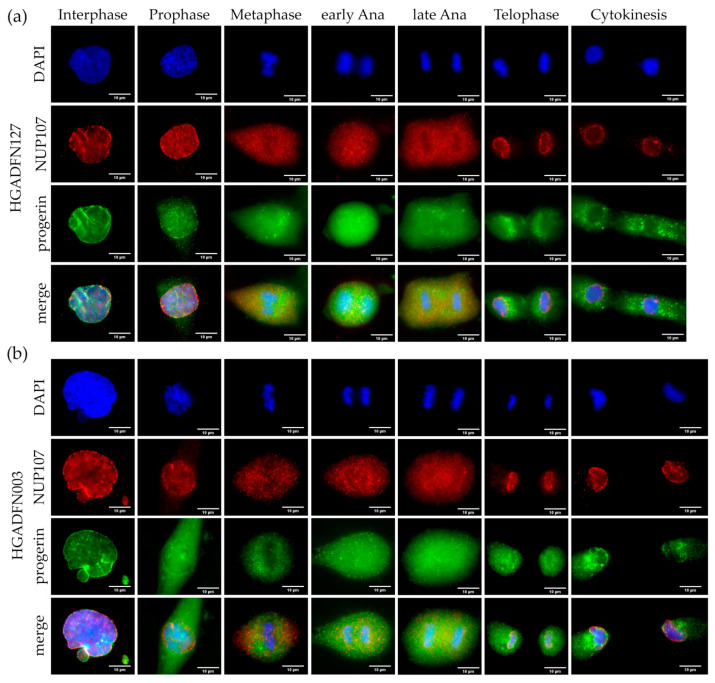
Progerin aggregates did not colocalize with NUP107 in mitotic Hutchinson-Gilford progeria syndrome (HGPS) cells. Immunocytochemistry of (**a**) HGADFN127 and (**b**) HGADFN003 fibroblasts using αi-NUP107 and α-progerin antibody, counterstained with DAPI. In metaphase to cytokinesis, progerin forms aggregates in the cytoplasm of mitotic HGPS cells, which are absent in control cells. Recruitment of NUP107 to late anaphase/telophase chromosomes is not delayed in HGPS cells. n ≥ 3.

To identify potential alterations, we tracked progerin and NUP107 from prophase to cytokinesis in two HGPS cell lines (Figure 2a,b). In interphase, we observed the characteristic NE invaginations, in which progerin and NUP107 were collected (Figure 2a,b). In metaphase HGPS cells, progerin formed aggregates close to metaphase chromosomes, whereas NUP107 was evenly distributed in the cytoplasm (Figure 2a,b). In early and late anaphase, progerin displayed aggregates unlike NUP107 (Figure 2a,b). By late anaphase to telophase, NUP107 was assembled at the reforming NE as expected. We detected no NUP107 aggregates colocalizing with progerin at any point and NUP107 was completely recruited to the NE by the end of telophase (Figure 2a,b and Tables S1 and S2). In contrast progerin was still aggregated in the cytoplasm of cytokinetic cells, with only faint rim-like staining detectable at the NE (Figure 2a,b). To confirm that NPC reassembly was not affected by progerin, we examined three additional NUPs from different NPC subunits: ELYS, NUP153, and POM121.

### 3.2. Post-Mitotic Seeding of the Nuclear Pore Complex on Anaphase Chromosomes is Not Affected in HGPS Cells

**Figure 3:**
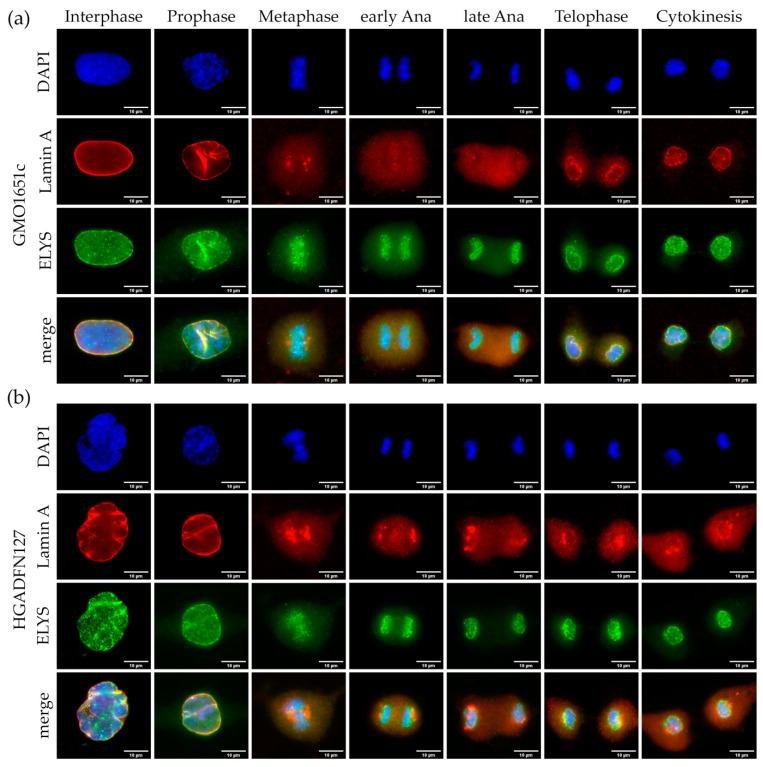
Seeding of NPCs by ELYS on anaphase chromosomes was not affected in HGPS. Immunocytochemistry of (**a**) control (GMO1651c) and (**b**) HGPS (HGADFN127) fibroblasts using α-ELYS and α-LA antibody, counterstained with DAPI. NPCs cluster in interphase HGPS cells (**b**), unlike the evenly spread out punctate pattern in interphase control cells (**a**). Recruitment of ELYS to anaphase chromosomes is not affected in HGPS cells when compared to control. No defect in ELYS localization can be observed in HGPS from prophase to cytokinesis. n ≥ 3.

Post-mitotic reassembly of NPCs is initiated by seeding of ELYS on anaphase chromosomes [15,16], followed by recruitment of the remainder of the NUP107-160 complex [16,17]. To determine, if differences in the initial step of NPC assembly occur, we evaluated the localization of ELYS and LA throughout the cell cycle (Figure 3 and Figure S1). Notably, the anti-lamin A antibody used in this study also recognizes progerin.

In interphase control cells, ELYS was evenly distributed at the NE exhibiting a classical punctate NPC pattern (Figure 3a and Figure S1a), whereas in HGPS cells, it localized and accumulated at the characteristic NPC clusters within the NE invaginations, as previously reported [5] (Figure 3b and Figure S1b). In both control and HGPS cells, we detected ELYS foci concentrated on metaphase chromosomes presenting a punctate pattern (Figure 3a,b and Figure S1a,b). In early anaphase, ELYS remained attached to the segregating chromosomes (Figure 3a,b and Figure S1a,b). In late anaphase, ELYS started to form a rim-like pattern around the chromosome mass (Figure 3a,b and Figure S1a,b). This continued until cytokinesis, where only a marginal ELYS background signal was detected in the cytoplasm of control and HGPS cells (Figure 3a,b and Figure S1a,b). The recruitment and assembly of ELYS remained unperturbed in HGPS, unlike LA (Figure 3b and Figure S1a,b). LA generally assembles at a later point in mitosis [23,35] and did not appear as a rim until telophase in control cells (Figure 3a). During metaphase until late anaphase, LA was detected as a diffuse cloud in the cytoplasm of control cells (Figure 3a and Figure S1a). In contrast, in HGPS cells, LA formed aggregates at the core region of metaphase chromosomes that remained present during anaphase (Figure 3b and Figure S1b). In HGPS cells, we detected a weaker LA rim staining around telophase DNA, than in control cells (Figure 3a,b and Figure S1a,b). Hence, in HGPS cells, LA aggregates remained detectable in the cytoplasm during telophase. In control cytokinetic cells, LA was nearly fully assembled in a clear rim, while in HGPS cells, the LA rim was barely detectable and large aggregates remained trapped in the cytoplasm (Figure 3a,b and Figure S1a,b).

In cytokinetic HGPS cells, NPC clustering was negligible when compared to interphase, with no large NPC aggregates detectable (Figure 3a,b and Figure S1a,b). Overall, we did not detect a difference in ELYS recruitment and localization between HGPS and control cells during mitosis. As initial seeding of ELYS during anaphase was not affected in HGPS, compared with LA, and no further alterations were detected during subsequent mitotic stages, we concluded that progerin does not demonstrate a detrimental effect on ELYS assembly in mitotic HGPS cells (Tables S1 and S2).

### 3.3. Post-Mitotic NPC Integration of Nuclear Basket Subunit NUP153 and NUP107 is Not Delayed in HGPS

**Figure 4:**
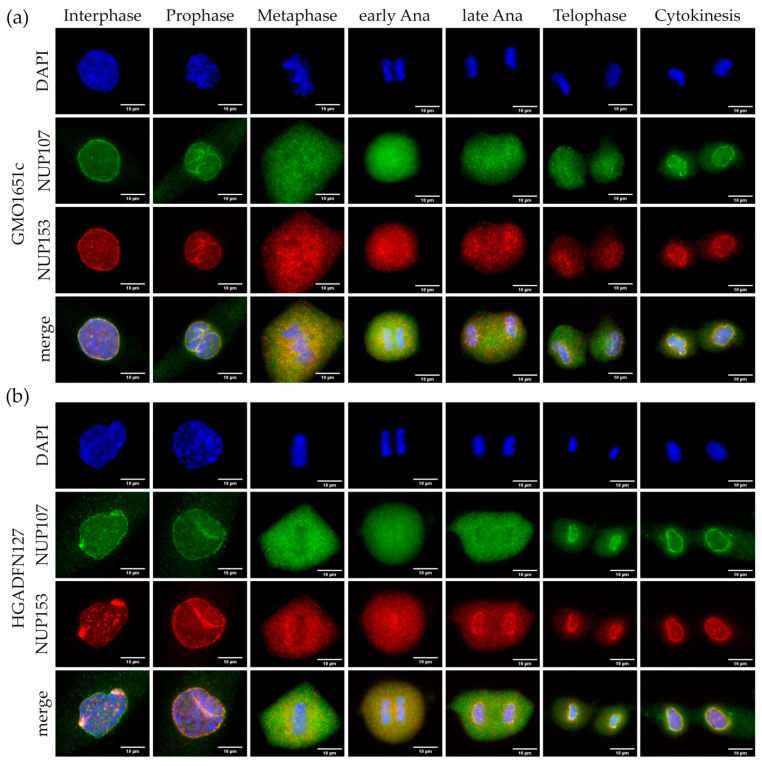
Recruitment of basket nucleoporin NUP153 and scaffold nucleoporin NUP107 was not affected in HGPS. Immunocytochemistry of (**a**) control (GMO1651c) and (**b**) HGPS (HGADFN127) fibroblasts using α-NUP107 and α-NUP153 antibodies, counterstained with DAPI. NPCs cluster in interphase HGPS cells (**b**), unlike the evenly distributed punctate pattern in control interphase (**a**). Recruitment of NUP153 to anaphase chromosomes is not affected in HGPS cells when compared to control. No defect in NUP153 or NUP107 localization can be observed in HGPS from prophase to cytokinesis. n ≥ 3.

NUP153 is one of the first members to be recruited to early anaphase chromosomes, following ELYS and the NUP107-160 complex [18,19,20], and its recruitment occurs before LB1 and LA [23,35]. Therefore, we examined NUP153 localization throughout mitosis in comparison to NUP107 in both HGPS and control cells, to rule out a potential defect in NPC basket assembly (Figure 4 and Figure S2).

Both control and HGPS showed typical NE invaginations during prophase, formed by a collapsing NE as previously reported [36] (Figure 4a,b and Figure S2a,b). Both NUP107 and NUP153 collected in the folds of the collapsing NE in prophase, before the NPCs were dissociated completely (Figure 4a,b and Figure S2a,b). NUP107 diffused into the cytoplasm faster than NUP153, which was not surprising, as NUP153 is localized inside the nucleus (Figure 4a,b and Figure S2a,b).

In both control and HGPS, NUP153 and NUP107 were evenly dispersed in the cytoplasm (Figure 4a,b and Figure S2a,b). However, NUP153 started to be recruited to the core region of the chromosome mass in early anaphase, while NUP107 remained diffused (Figure 4a,b and Figure S2a,b). In late anaphase, NUP153 started to surround the segregating chromosomes, while NUP107 remained mostly diffused throughout the cytoplasm (Figure 4a,b and Figure S2a,b). In telophase of HGPS and control cells, NUP153 exhibited a rim-like staining with barely detectable amounts in the cytoplasm (Figure 4a,b and Figure S2a,b). In contrast, most of NUP107 remained in the cytoplasm and only showed a weak rim-like staining indicating that its recruitment at the NE occurred after NUP153 (Figure 4a,b and Figure S2a,b). For both NUP153 and NUP107, the cytokinetic cells exhibited an even and overlapping rim signal, with no remaining signal detected in the cytoplasm (Figure 4a,b and Figure S2a,b). These observations indicated that similar to ELYS distribution, no aggregates or delayed assembly of NUP153 and NUP107 were observed in HGPS compared to control cells (Tables S1 and S2).

### 3.4. SUN1 Aggregates Do Not Affect the Recruitment of POM121 to Assembling NPCs

Following the seeding of NPCs by ELYS and the Y-complex, the transmembrane NUPs, POM121, and NDC1, are recruited to the reforming NE between early and late anaphase [16,21,22]. Given that SUN1 and POM121 function in concert for interphase NPC assembly [28,29], we hypothesized that SUN1 influences POM121 recruitment to the reforming NE during mitosis. SUN1 distribution is altered during mitosis in HGPS cells with aggregates colocalizing with progerin [8,31]. Therefore, we used immunocytochemistry to follow POM121 and SUN1 localization throughout mitosis, both of which localize to the endoplasmic reticulum (ER) during mitosis [23,31]. We could not perform a co-stain of POM121 and SUN1, as both available antibodies are from the same species (rabbit), and no alternative options were available.

**Figure 5:**
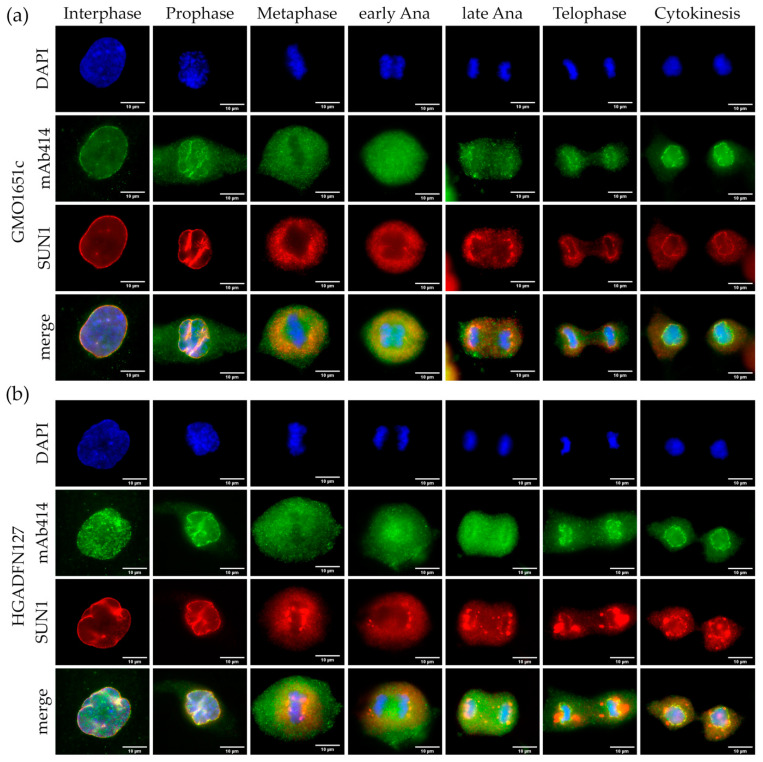
SUN1 aggregates did not colocalize with mAb414. Immunocytochemistry of (**a**) control (GMO1651c) and (**b**) HGPS (HGADFN127) fibroblasts using mAb414 and α-SUN1 antibodies, counterstained with DAPI. NPCs cluster in interphase HGPS cells (**b**) and the aggregates colocalize with SUN1 aggregates. In metaphase to cytokinesis, SUN1 forms aggregates in the ER of mitotic HGPS cells, which are absent in control cells. Recruitment of mAb414 to late anaphase/telophase chromosomes is not affected in HGPS cells. n ≥ 3.

**Figure 6:**
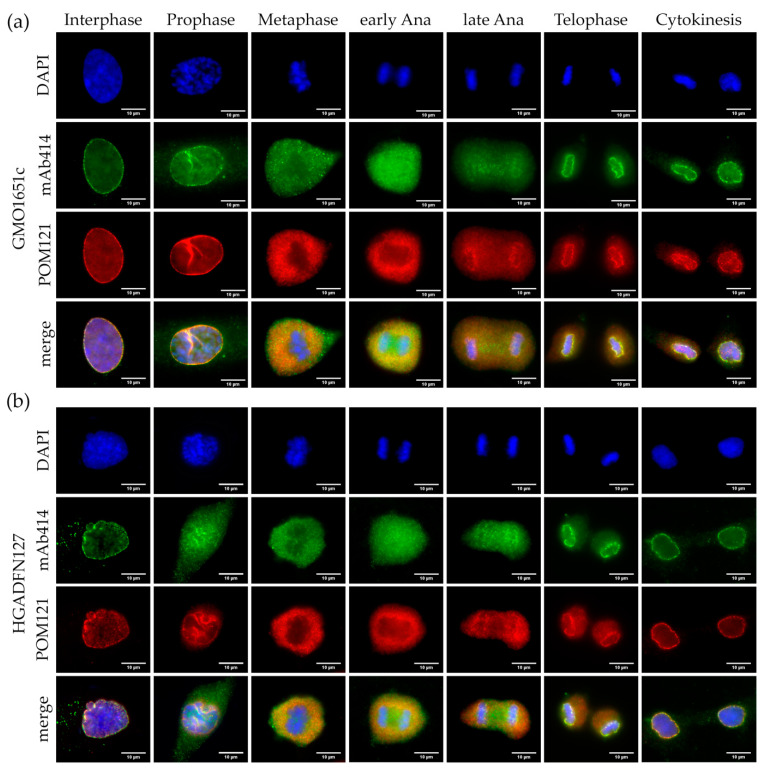
Unlike SUN1, POM121 did not form aggregates in HGPS. Immunocytochemistry of (**a**) control (GMO1651c) and (**b**) HGPS (HGADFN127) fibroblasts using mAb414 and α-POM121 antibody, counterstained with DAPI. POM121 and mAb414 cluster in invaginations of interphase HGPS cells. Neither the localization nor recruitment of mAb414 and POM121 were affected in mitotic HGPS cells when compared to control. n ≥ 3.

Consequently, we performed double labeling of either SUN1 (Figure 5 and Figure S3) or POM121 (Figure 6 and Figure S4) with mAb414 antibody recognizing NUP62, NUP153, NUP358, and NUP214 [37,38]. We expected mAb414 to be recruited after POM121 and to collect around the chromosomes in a more diffuse pattern.

In interphase control cells, SUN1 formed an even rim-like stain (Figure 5a and Figure S3a), while appearing trapped in the invaginations of interphase HGPS cells (Figure 5b and Figure S3b). In prophase, the repartitioning of SUN1 in HGPS did not differ from control cells, collecting in the folds of the collapsing NE (Figure 5a,b and Figure S3a,b). The mAb414 signal began dissociating from the prophase NE and became diffuse in the cytoplasm (Figure 5a,b and Figure S3a,b). SUN1, as a transmembrane protein, is redistributed in the ER during mitosis [31] and was excluded from the area of metaphase chromatin in both HGPS and control cells (Figure 5a,b and Figure S3a,b). However, in HGPS cells, SUN1 was not evenly distributed, and formed large aggregates close to metaphase chromosomes (Figure 5b and Figure S3b). During early to late anaphase in control cells, SUN1 started to assemble at the core region of the segregating chromosomes, enclosing them by telophase (Figure 5a and Figure S3a). In HGPS cells, SUN1 started assembling at the core region too. However, it also localized at sites of distinct aggregates in the cytoplasm (Figure 5b and Figure S3b, Tables S1 and S2). This was not the case for NUPs labeled by mAb414, which began depositing around chromosomes in late anaphase in both control and HGPS cells, with no visible mAb414 aggregates (Figure 5a,b and Figure S3a,b). In telophase SUN1 formed a smooth rim around the chromosomes in control cells, not observed in HGPS cells (Figure 5a,b and Figure S3a,b). In HGPS cells, the large SUN1 positive aggregates were mostly localized at the core region from metaphase to anaphase and remained present in the cytoplasm during cytokinesis (Figure 5b and Figure S3b, Tables S1 and S2). In telophase, the mAb414 signal showed the same distribution for both control and HGPS, with the classic punctate pattern surrounding the chromosomes (Figure 5a,b and Figure S3a,b). Notably, not all the NUPs labeled by mAb414 are completely incorporated in telophase or cytokinesis, and the cytoplasmic filament NUPs of the NPC do not completely assemble until G1 [18]. In the cytoplasm, the residual mAb414 signal was stronger in cytokinetic HGPS cells, which might be attributed to the delay of NE reformation in HGPS cells [8] (Figure 5b and Figure S3b).

Next, we examined the localization of POM121 in relation to mAb414 in both control and HGPS cells (Figure 6 and Figure S4). Similar to mAb414 distribution, POM121 was clustered in the invaginations of interphase HGPS cells (Figure 6b and Figure S4b), while it was evenly distributed in control cells (Figure 6a and Figure S4a).

In both HGPS and control cells, POM121 appeared to dissociate from the NE later than the NUPs labeled by mAb414 (Figure 6a,b and Figure S4a,b). Furthermore, POM121 was recruited in collapsed sites of the NE to a greater extent than that observed with mAb414 (Figure 6a,b and Figure S4a,b). In metaphase cells, mAb414 was evenly dispersed in the cytoplasm, whereas POM121 was excluded from the region of the metaphase chromosomes, similarly to SUN1 (Figure 5a and Figure 6a,b, Figure S4a,b). In early anaphase, POM121 was detected around the chromosomes and mitotic spindle area, whereas the mAb414 signal was also present between the segregating chromosomes (Figure 6a,b and Figure S4a,b). POM121 formed a clear ring around the chromosomes starting in late anaphase, whereas the mAb414 rim signal was weaker (Figure 6a,b and Figure S4a,b). In telophase, the recruitment of both POM121 and mAb414 was nearly complete, with no visible clusters observed in either control or HGPS cells (Figure 6a,b and Figure S4a,b, Tables S1 and S2). Cytokinetic HGPS cells presented the same classical punctate NPC pattern, as observed in control cells (Figure 6a,b and Figure S4a,b).

Collectively, compared to SUN1, POM121 and NUPs detected by mAb414 showed no significant alterations or delayed recruitment to the reforming NE in the presence of progerin (Tables S1 and S2).

### 3.5. Relationship between Dysmorphic Nuclei and Replicative Senescence

We did not detect any obvious alterations in the distribution of the different components of the NPC during mitosis (Table S2). However, since we observed clustering in some interphase HGPS and control cells, we investigated the cellular state of the cells exhibiting NPC clusters.

**Figure 7:**
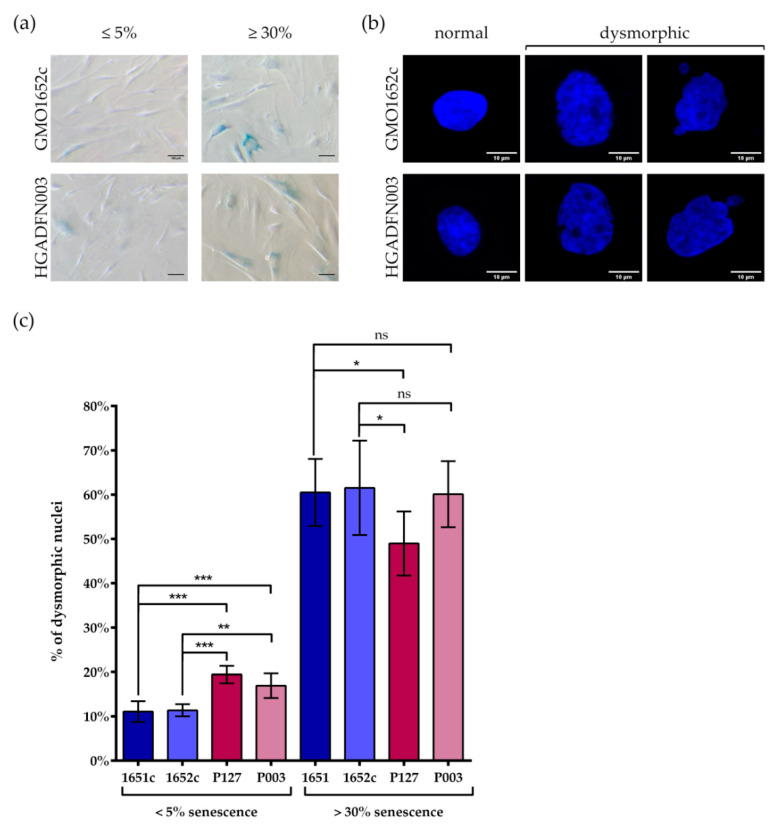
Number of dysmorphic nuclei increased with replicative senescence in both control and HGPS. (**a**) Representative images of SA-β-galactosidase stained cells. (**b**) Representative images of normal and dysmorphic nuclei counterstained with DAPI. (**c**) The number of dysmorphic cells in <5% and >30% control and HGPS fibroblasts, was determined by counting nuclei counterstained with DAPI. Comparisons within control or HGPS cell lines, with similar senescence, are not significant. At 5% senescence, HGPS passage numbers were ≤P18, control cells were ≤P21. At 30% senescence, control passages were ≥P25, HGPS were ≥P21. Values are presented as mean ± SD (n ≥ 3), not significant (ns) *p* > 0.05, * *p* < 0.05, ** *p* < 0.01, *** *p* < 001, one-way ANOVA with Tukey’s multiple comparison test.

In a recent study, we examined the similarities in cellular aging between HGPS and control cells [39]. We have shown that progerin accumulates in senescing cells during long-term culture [39]. As HGPS cells senesce at earlier passage numbers than control cells [39], we investigated control and HGPS cultures at senescence (SNS) index of ≤5% and ≥30%, defined by the number of cells positive for senescence associated β-Galactosidase (SA-β-Gal) (Figure 7a). By comparing control and HGPS cultures exhibiting a similar SNS index, we classified and determined the percentage of nuclei with normal ovoid nuclear shape and those presenting abnormal and large morphologies, considered dysmorphic (Figure 7b). Young HGPS cultures (≤5% SNS) contained significantly higher numbers of dysmorphic nuclei (~18%) than their control counterparts (~11%) (Figure 7c). However, in older cultures (≥30% SNS) the presence of dysmorphic nuclei in control and in HGPS cultures was relatively similar (Figure 7c). The number of dysmorphic nuclei increased three-fold (~58%), in all four-primary fibroblast strains analyzed during replicative senescence (Figure 7c).

Collectively, this study highlights that nuclear dysmorphism was prominent in young HGPS cultures relative to young controls; however, this difference was not observed in old cultures from both cell types. This indicated that progerin expression in young HGPS cells was the primary cause for nuclear abnormalities in shape and size, while other cellular processes may account for further nuclear dysmorphism in late control and HGPS cultures.

**Figure 8:**
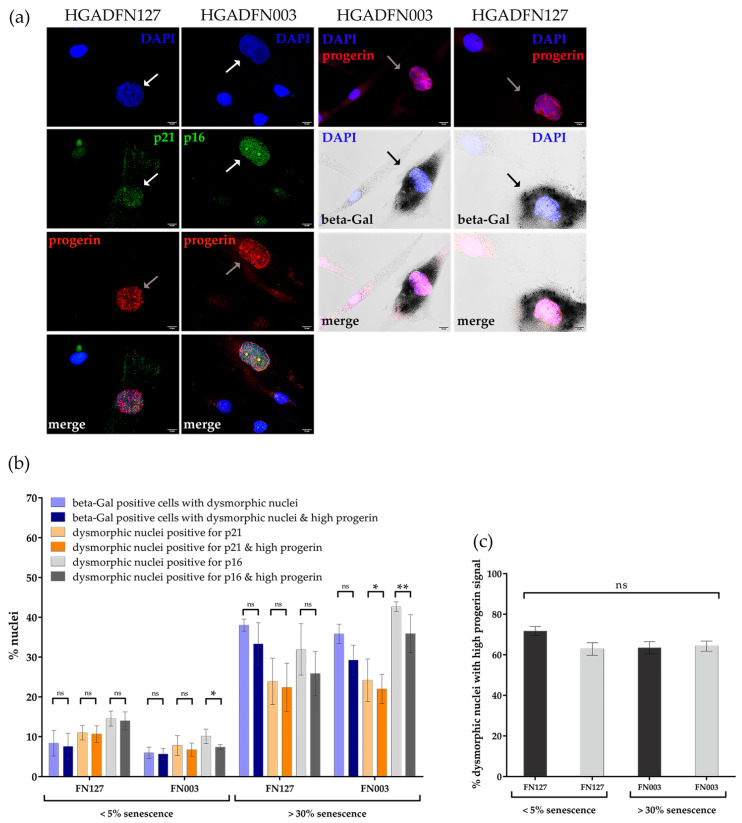
Cells with high levels of progerin were senescent. (**a**) Representative images of cells stained for α-progerin and α-p16/p21 or β-Gal, counterstained with DAPI. Arrows indicate nuclei with a high progerin signal and positive for either high p16 or p21 or beta-Gal. (**b**) The number of dysmorphic nuclei with strong progerin signal positive for SA-β-Gal and positive for p16 or p21 increased during replicative senescence. (**c**) The number of dysmorphic nuclei with strong progerin signal remained constant during replicative senescence. At 5% senescence, HGPS cells had a passage number ≤ P18 and at 30% senescence ≥ P21. Values are presented as mean ± SD (n ≥ 3), not significant (ns) *p* > 0.05, * *p* < 0.05, ** *p* < 0.01, (**b**,**c**) unpaired *t*-test, (**c**) one-way ANOVA with Tukey’s multiple comparison test.

To elucidate why old control and HGPS cultures (>30%SNS) exhibited a similar percentage of dysmorphic nuclei, we determined whether cells exhibiting nuclear abnormalities were senescent cells. Using immunocytochemistry, we screened for the expression of two known senescence markers p16^INK4A^ (p16) and p21 in parallel to progerin in HGPS cultures (Figure 8a) [40,41,42].

A majority of HGPS cells exhibiting a bright progerin-positive signal harbored p21, p16 and SA-β-Gal positive signals (Figure 8a). We further assessed the percentage of HGPS cells presenting a progerin-positive signal and senescence-positive markers (p21, p16 and SA-β-Gal) in young and old HGPS cultures (Figure 8b,c). Nuclei were scored with the following characteristics: dysmorphic, positive for one of the senescence markers, and a strong progerin signal (Figure 8b). Remarkably, more than 60% of dysmorphic nuclei present in young and old HGPS cultures were brightly progerin-positive, implying that these cells had accumulated high amounts of progerin (Figure 8c). Furthermore, the percentage of dysmorphic nuclei with a high progerin content and p16 positive signal was similar to the total percentile of dysmorphic nuclei positive for p16 in both early and late cultures, although their numbers were 3-fold higher in late cultures (Figure 8b). Next, the percentage of dysmorphic nuclei harboring high progerin levels and p21 positive signal were also similar to the total number of dysmorphic nuclei with p21 positive signals in young and old cultures (Figure 8b).

Notably, most young or old dysmorphic HGPS cells with positive SA-β-Gal signal also showed a bright progerin signal (Figure 8b). This increased by five-fold from young to old HGPS cultures, which was a significantly higher increase than that for p16 or p21 (Figure 8b). On comparing the four markers, SA-β-Gal scored less dysmorphic nuclei in young HGPS cells than the other senescence markers (p16/p21) (Figure 8b). In old HGPS cultures we scored similar numbers for all three SNS markers, therefore, the difference in fold-change could be attributed to SA-β-Gal being a late senescence marker.

Our results indicated that progerin is the main cause of increased nuclear abnormalities observed in young HGPS cultures compared with young controls. Additionally, the bright progerin signal was indicative of senescent cells, as these cells were also positive for any of the three senescence markers we analyzed (Figure 8b).

We also analyzed the two SNS markers p16 and p21 in young and old control cultures, compared with HGPS cultures (Figure S5a,b). As observed with HGPS cells, most young and old dysmorphic control nuclei were positive for p16 or p21, with a concomitant increase in their numbers from young to old cultures (Figure S5b). The percentage of senescent dysmorphic nuclei increased from 3- fold to 6-fold in young to old control cultures, according to the senescence marker analyzed (Figure S5b). Notably, in old control cultures, the percentage of dysmorphic senescent nuclei was similar to that observed in old HGPS cultures with a similar SNS index (Figure S5b).

Our results indicated that the number of dysmorphic and senescent nuclei increased with replicative senescence in all cell types, as evidenced by the three distinct senescence markers (Figure 8b and Figure S5b). Furthermore, we demonstrated that the severity of nuclear abnormalities was dependent on progerin levels in cells from young HGPS cultures. However, in old control cultures, the similarly increased number of dysmorphic nuclei was probably due to nuclear defects associated with processes inherent to replicative senescence. Collectively, our findings demonstrated that nuclear buildup of progerin not only leads to nuclear abnormalities, but is a marker of HGPS senescent cells.

### 3.6. NPC Clustering in Interphase Control and HGPS Cells is Associated with Replicative Senescence

**Figure 9:**
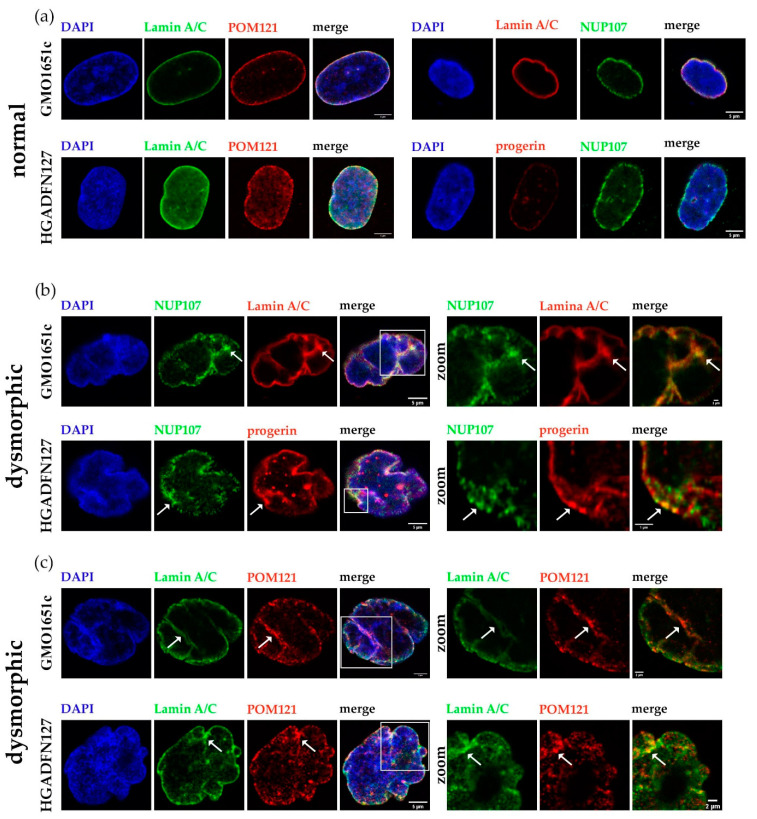
NPCs clustered in folds of dysmorphic nuclear envelopes and co-localized with progerin/Lamin A aggregates trapped at the nuclear membrane. (**a**) Representative images of normal control (GMO1651c) and HGPS (HGADFN127) nuclei stained with α-NUP107/POM121/LA/progerin antibodies, counterstained with DAPI. (**b**) Representative images of dysmorphic control and HGPS nuclei, stained with α-NUP107 and α-LA/C or progerin antibodies, counterstained with DAPI. (**c**) Representative images of dysmorphic control and HGPS nuclei, stained with α-POM121 and LA antibodies, counterstained with DAPI. Arrows indicate regions with clustered NPCs, outlines indicate zoomed in section of nuclei. All images are taken from z-stack and are provided as X-Z view. Zoom factor: (**b**) GMO1651c 15x, HGADFN127 25x; (**c**) GMO1651c 9x, HGADFN127 12x.

Given that we observed NPC clustering in interphase control cells with nuclear abnormalities, we examined whether this occurred during replicative senescence as a consequence of nuclear lamina alterations. To confirm that our observation of NPC clusters were not nuclear foci, but rather trapped NPCs in the nuclear envelope invagination, we imaged z-stacks of HGPS and control nuclei (Figure 9). We probed a transmembrane NUP (POM121) and a member of the inner/outer ring (NUP107) in combination with lamin A/C or progerin to detect the localization of NPCs in normal and dysmorphic nuclei (Figure 9a–c).

In normal, ovoid control and HGPS nuclei, both POM121 and NUP107 were evenly distributed across the NE and the progerin signal was weak (Figure 9a). However, in dysmorphic control and HGPS nuclei, the distribution of NPC was no longer even and accumulated to NE folds or blebs (Figure 9b,c (arrows)). Additionally, NUP107 clusters co-localized with a strong progerin signal (Figure 9b, arrows), highlighted in the zoomed image, confirming that progerin accumulation influenced NPCs distribution.

**Figure 10:**
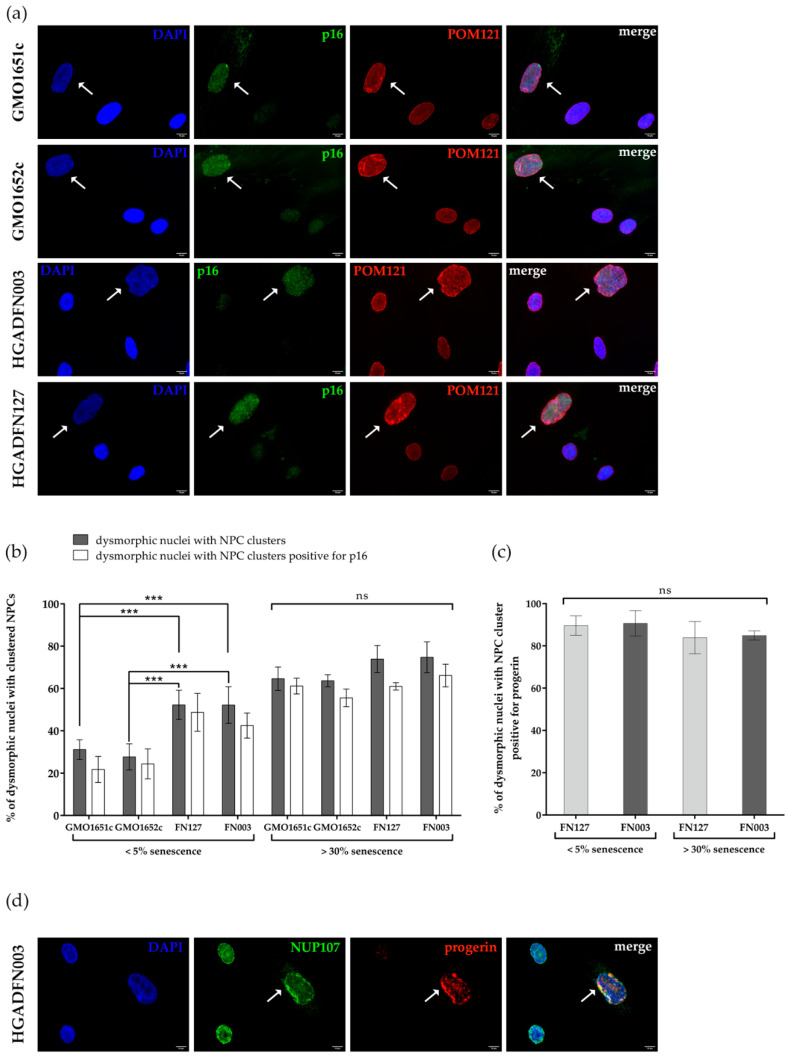
Number of dysmorphic nuclei with clustered NPCs increased with replicative senescence. (**a**) Representative images of cells stained with α-p16 and α-POM121 antibodies, counterstained with DAPI. White arrows indicate dysmorphic nuclei with clustered NPCs and elevated p16. (**b**) The numbers of dysmorphic nuclei with clustered NPCs, in cultures ≤5% senescence and ≥30% senescence of control and HGPS cells, were determined by counting dysmorphic nuclei positive for clustered NPCs and p16 positive signal, counterstained with DAPI. Differences between young and old cells in each cell line are significant. (**c**) The numbers of dysmorphic nuclei with clustered NPCs and progerin positive. On average, 87% of dysmorphic HGPS nuclei with clustered NPCs are positive for progerin. No significant difference could be determined depending on replicative senescence. (**d**) Representative images of HGADFN003 stained with α-NUP107 and α-progerin antibodies, counterstained with DAPI. White arrows indicate NUP107 clusters overlapping with strong progerin signal. At 5% senescence, HGPS cell passages were ≤P18, and control cells were ≤P21. At 30% senescence control passages were ≥P25, HGPS were ≥P21. Values are presented as mean ± SD (n ≥ 3), ns *p* > 0.05, *** *p* < 001, (**b**) two-way ANOVA with Tukey’s multiple comparison test, (**c**) one-way ANOVA with Sidak’s multiple comparison test.

As clustering of NPCs occurred mostly in interphase dysmorphic nuclei with blebs or folds, we evaluated the statistics of this phenomenon and determined, if these dysmorphic cells were senescent. As SA-β-Gal staining was not compatible with the immunofluorescence detection of NPCs, we used p16 staining of dysmorphic nuclei. We used p16 in combination with the transmembrane NUP POM121 to investigate the relationship between senescence, NPC clustering, and progerin (Figure 10a). In dysmorphic nuclei, POM121 was clustered in the NE folds in both control and HGPS cells (Figure 10a, white arrows). These p16 positive nuclei were frequently enlarged, presenting a DNA-DAPI signal less intense than normal ovoid nuclei (Figure 10a).

In young control cultures (<5% SNS) approximately 29% of dysmorphic nuclei exhibited clustered NPCs, while 52% were present in HGPS counterparts (Figure 10b). In contrast, an average of 69% of dysmorphic nuclei exhibited POM121 clusters in old cultures (>30%SNS), in both HGPS and control cells (Figure 10b). Similar to nuclear dysmorphism (Figure 7c), a significant difference in POM121 clustering was detectable exclusively in young cultures: 29% in control vs. 52% in HGPS, whereas old cultures displayed a similar amount of clustering (~69%) (Figure 10b). Additionally, most HGPS and control dysmorphic nuclei with clustered POM121 exhibited an elevated p16 signal (Figure 10a,b), suggesting that NPC clustering is linked to replicative senescence. Furthermore, the clustering of NPCs was associated with the tendency of NPC proteins (POM121 and NUP153) to increase during replicative senescence in both cell types (Figures S6 and S7).

Our previous findings indicated that progerin expression and accumulation were the main causes of nuclear abnormalities in young HGPS cultures (Figure 8c and Figure S6). Therefore, we analyzed the number of dysmorphic nuclei with clustered NPCs exhibiting a strong progerin signal in young and old HGPS cultures (Figure 10c,d). We used progerin in combination with NUP107 and observed that on average 87% of dysmorphic nuclei with clustered NUP107 presented a strong progerin signal in both young and old HGPS cultures (Figure 10c,d).

In conclusion, NPC clustering in HGPS cells occurred concomitantly with progerin nuclear buildup. Compared to young controls, the higher number of dysmorphic nuclei with POM121 clusters in young HGPS cultures was attributed to progerin expression. In old HGPS and control cultures, the similar percentages of dysmorphic nuclei with POM121 clusters were likely associated with increased NE defects driven by replicative senescence.

## 4. Discussion

Nuclear import and the Ran gradient are both disturbed in progeria, and potentially caused by NPC clustering [43,44,45,46]. In dysmorphic HGPS nuclei, NPCs collect in the NE folds, which could restrict access to transport receptors and cargo. To uncover the underlying mechanism of clustering, we investigated the localization of multiple NUPs in mitotic and aging cells.

In the current study, we demonstrated that the post-mitotic assembly of the NPC was not affected by progerin. One explanation could be the timing of NE reassembly, combined with low progerin levels in young mitotically active cells. We screened multiple NUPs from different subunits of the NPC, observing that none of them demonstrated delayed recruitment, nor did they aggregate in the cytoplasm of mitotic HGPS cells. Seeding of the NPC by ELYS is initiated during early anaphase [47], and we failed to detect a recruitment delay for any NUP part of the NPC subcompartments examined.

Localization of POM121 and NUP153 was especially interesting, as POM121 is a transmembrane nucleoporin, and NUP153 is a known interactor of lamins [48,49]. Previously, we had reported that progerin negatively affects numerous nuclear membrane proteins during NE reassembly following mitosis, including LB1, emerin, and SUN1 [8]. Toward the end of mitosis, progerin forms aggregates, coinciding with LB1, emerin, and SUN1 aggregates that remained in the endoplasmic reticulum [8]. Therefore, we expected to detect POM121 and NUP153 aggregates; however, this was not observed. A possible explanation for the lack of NP153/POM121 aggregates could be that POM121 and NUP153 recruitment to the reforming NE occurs before nuclear lamins [23]. Previous studies have indicated that NUP153 and POM121 concentrate at the chromosomes before LB1, while mAb414 appears after LB1 and before LA/LC [23,35]. Consequently, assembly should occur as follows: NUP153, POM121, LB1, some NUPs detected by mAb414 antibody, and LA/C [23,35]. Therefore, progerin might not interfere with post-mitotic NPC assembly, because reassembly begins before LA/C and progerin are involved.

As the distribution of NPCs was not affected by changes in post-mitotic assembly, NPC clustering can probably be explained through altered de novo interphase assembly or distribution. Interphase NPC assembly proceeds via an inside-out-extrusion, requiring the fusion of the inner and outer membranes of the NE [50]. Several components are known to be necessary: RanGTP [51], recruitment of NUP107-160 by NUP153 [52], the membrane-curvature sensing domain of NUP133 [53], presence of POM121 [29] and SUN1 [28]. In cells that contain high levels of progerin, the NE is stiff and access to the membrane is likely restricted. Additionally, SUN1 strongly interacts with progerin in mitotic HGPS cells and collects in the NE along with progerin [8]. The presence of progerin limits SUN1 mobility in the NE [54], preferentially interacting with farnesylated LA (preLA) [30]. Therefore, it is plausible that progerin aggregates block access to the INM, and restricted SUN1 mobility prevents even distribution of NPCs during interphase assembly. Another essential component of interphase assembly is the nucleoplasmic RanGTP [51] and the Ran gradient is disturbed in HGPS [44,45]. If no new NPCs are inserted into the nuclear membrane of aging cells, owing to the lack of nucleoplasmic RanGTP, the remaining pores might become trapped by the progerin/SUN1 aggregates, and therefore cluster.

Hence, the combination of progerin physically blocking access to the INM, limited mobility of SUN1, and a deregulated Ran gradient could prevent the even distribution of NPCs and cause NPCs to remain trapped and clustered in the NE folds.

In control cells with clustered NPCs, dysmorphic nuclei lack progerin and SUN1 levels are much lower than in HGPS. Thus, the question remains as to why NPCs cluster without progerin stiffening the NE. One rationale for clustering in senescent nuclei could be the deregulation of NE proteins during senescence. Reduced LB1 levels are considered a marker for senescent cells [42] and can result in uneven NPC distribution [55]. Furthermore, high levels of LA/C restrict the uniform distribution of NPCs, and low LA/C levels reduce the number of so-called pore-free islands [56]. Reduced LB1 levels could cause NE stiffening, similar to progerin, and as a result, NPCs could end up trapped in folds of dysmorphic control nuclei.

Our results indicate that the integrity of the nuclear lamina appears to be vital for proper NPC distribution in control and HGPS cells, and progerin increases the tendency of NPCs cluster formation in young HGPS cultures when compared to control cells. Moreover, replicative senescence affects the integrity of the nuclear envelope and lamina. Therefore, preventing HGPS cells from entering premature senescence could be an option to avert these alterations.

Additionally, we demonstrated that a strong progerin signal is directly linked to cellular senescence in HGPS cells. Therefore, progerin not only disrupts the nuclear lamina but causes further disruptions through premature senescence. Increased NE stiffness and premature senescence caused by progerin could be ameliorated by eliminating progerin. One current strategy for treating HGPS is to increase autophagy to remove progerin [34,39,57,58,59]. We recently discovered that the JAK/STAT inhibitor baricitinib decreases progerin levels and reduces premature senescence in HGPS cells [39]. Consequently, it could be postulated that baricitinib could additionally ameliorate or prevent NPC clustering and deregulation of the Ran gradient, by enhancing progerin clearance and delaying premature replicative senescence.

## Data Availability

Data is contained within the article or supplementary material.

## Supplementary Materials

The following are available online at https://www.mdpi.com/2073-4409/10/1/153/s1, Figure S1: Seeding of NPCs, by ELYS on anaphase chromosomes was not affected in HGPS. Figure S2: Recruitment of basket nucleoporin NUP153 and scaffold nucleoporin NUP107 were not affected in HGPS. Figure S3: SUN1 aggregates did not colocalize with mAb414. Figure S4: Unlike SUN1, POM121 does not form aggregates in HGPS. Figure S5: Evaluation of senescence markers. Figure S6: Evaluation of NUP153, POM121, SUN1 and Lamin A/C proteins in control and HGPS cellular extracts. Figure S7 Full-length scan of western blots from Figure S6. Table S1: Number of mitotic cells analyzed. Table S2: Frequency of mislocalized LA, SUN1 and NPC proteins in mitotic cells.
