# Supplementary material for: Nuclear Pore Complexes Cluster in Dysmorphic Nuclei of Normal and Progeria Cells during Replicative Senescence
Source: Cells. 2021 Jan 14;10(1):153. doi: 10.3390/cells10010153 (PMC7828780; doi:10.3390/cells10010153)
Supplement: Supplementary file 1 [file cells-10-00153-s001.pdf]

cells

MDPI

# Nuclear Pore Complexes Cluster in Dysmorphic Nuclei of Normal and Progeria Cells during Replicative Senescence


**Jennifer M. Röhrl, Rouven Arnold and Karima Djabali ***

Epigenetics of Aging, Department of Dermatology and Allergy, TUM School of Medicine, Technical University of Munich (TUM), 85748 Garching, Germany; jennifer.roehrl@tum.de (J.M.R.); rouven.arnold@tum.de (R.A.)

* Correspondence: djabali@tum.de


## Supplementary Figures

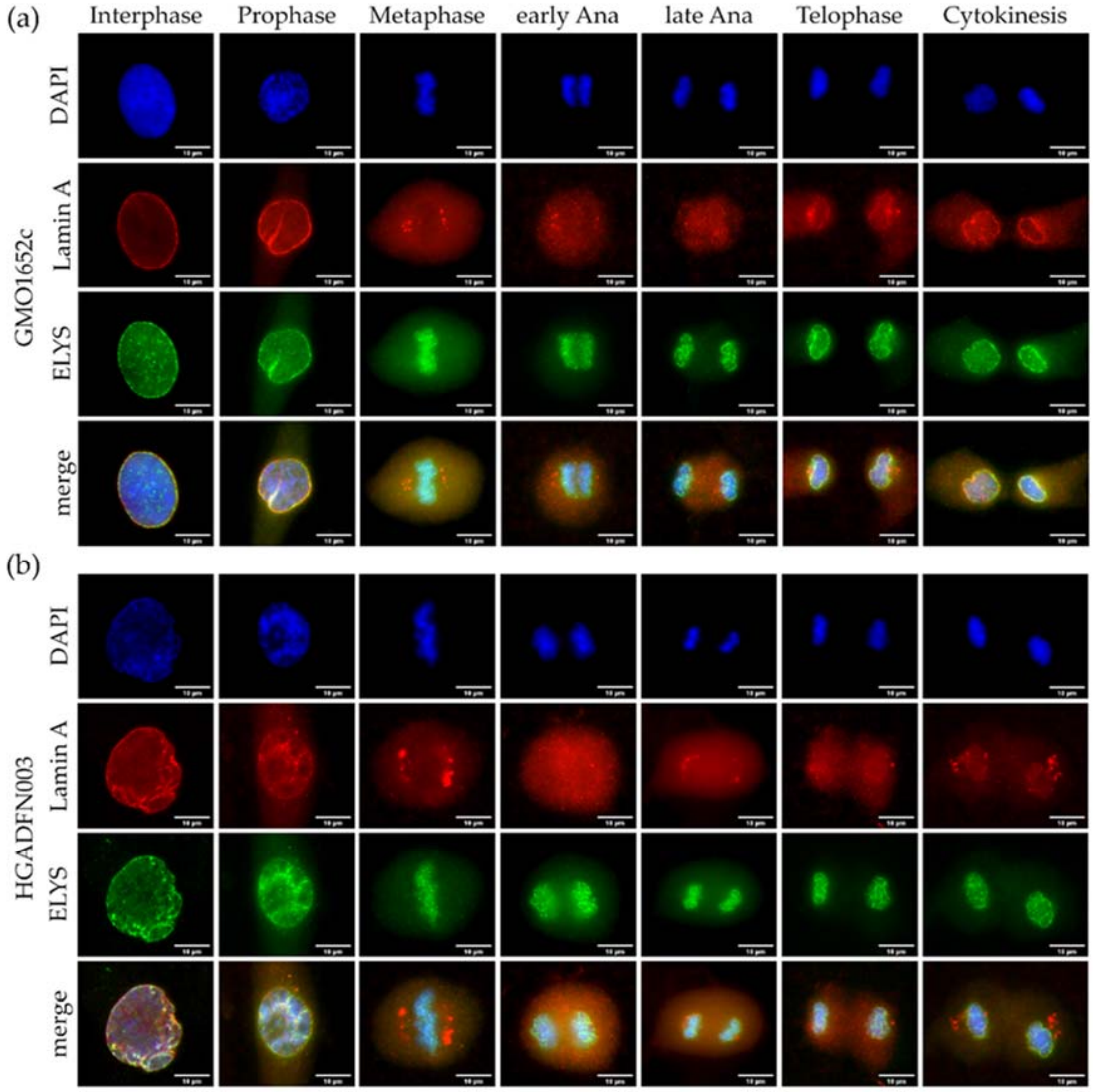


**Figure S1:** Seeding of NPCs, by ELYS on anaphase chromosomes was not affected in HGPS. Immunocytochemistry of (**a**) control (GMO1652c) and (**b**) HGPS (HGADFN003) fibroblasts using $\alpha$-ELYS and $\alpha$-LA antibody, counterstained with DAPI. NPCs cluster in interphase HGPS cells (**b**), unlike the evenly distributed punctate pattern in interphase control cells (**a**). Recruitment of ELYS to anaphase chromosomes is

not affected in HGPS cells, compared to control. No defect in ELYS localization can be observed in HGPS from prophase to cytokinesis. NPC, nuclear pore complex; HGPS, Hutchinson-Gilford progeria. n ≥3.

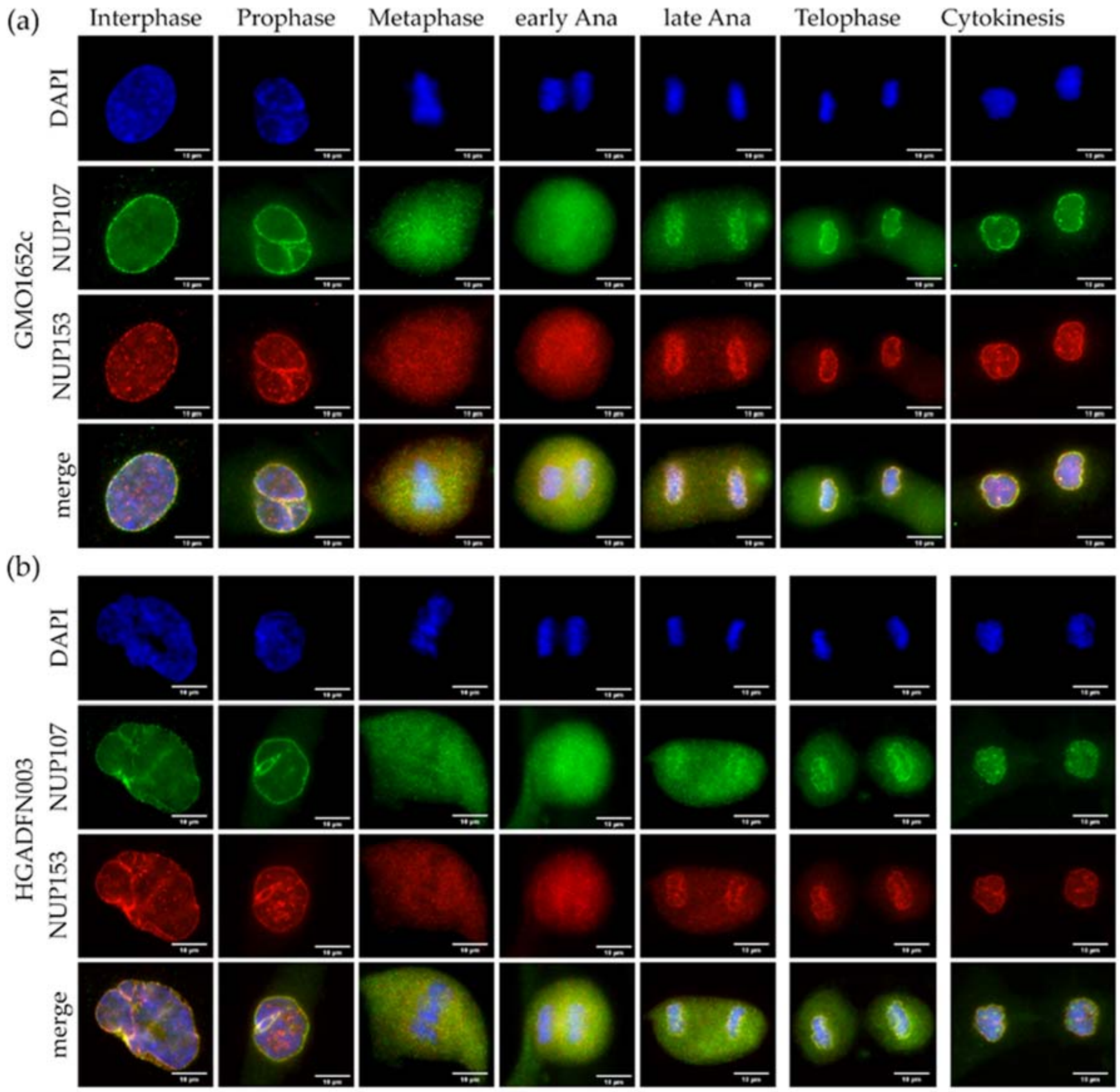


**Figure S2:** Recruitment of basket nucleoporin NUP153 and scaffold nucleoporin NUP107 were not affected in HGPS. Immunocytochemistry of (**a**) control (GMO1652c) and (**b**) HGPS (HGADFN003) fibroblasts using $\alpha$-NUP107 and $\alpha$-NUP153 antibodies, counterstained with DAPI. NPCs cluster in interphase HGPS cells (**b**), unlike the evenly spread out punctate pattern in control interphase (**a**). Recruitment of NUP153 to anaphase chromosomes is not affected in HGPS cells, compared to the control. No defect in NUP153 or NUP107 localization can be observed in HGPS from prophase to cytokinesis. n ≥3.

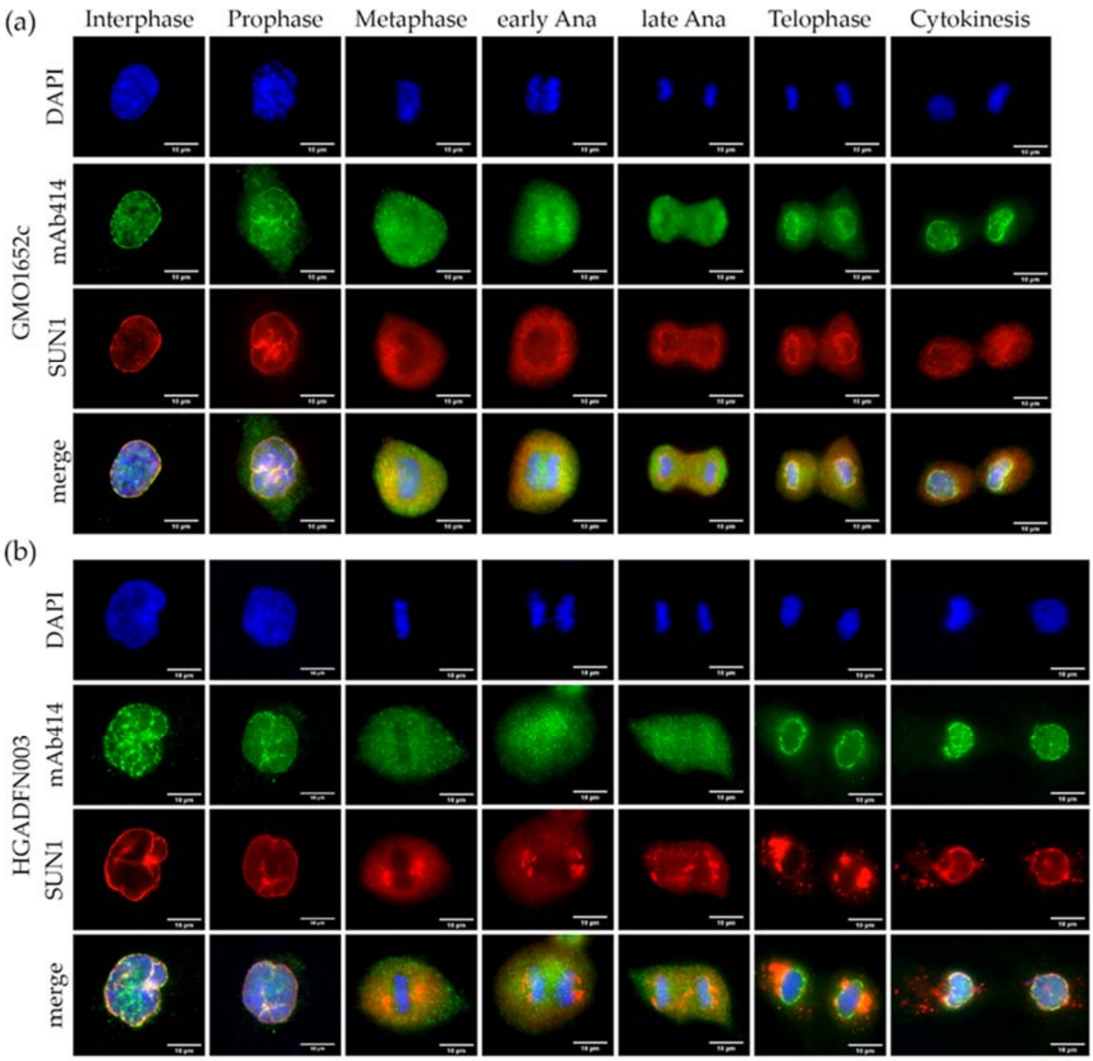


**Figure S3:** SUN1 aggregates did not colocalize with mAb414. Immunocytochemistry of (**a**) control (GMO1652c) and (**b**) HGPS (HGADFN003) fibroblasts using mAb414 and $\alpha$-SUN1 antibodies, counterstained with DAPI. In metaphase to cytokinesis, SUN1 forms aggregates in the cytoplasm of mitotic HGPS cells, absent in control cells. Recruitment of mAb414 to late anaphase/telophase chromosomes is not affected in HGPS cells. n ≥3.

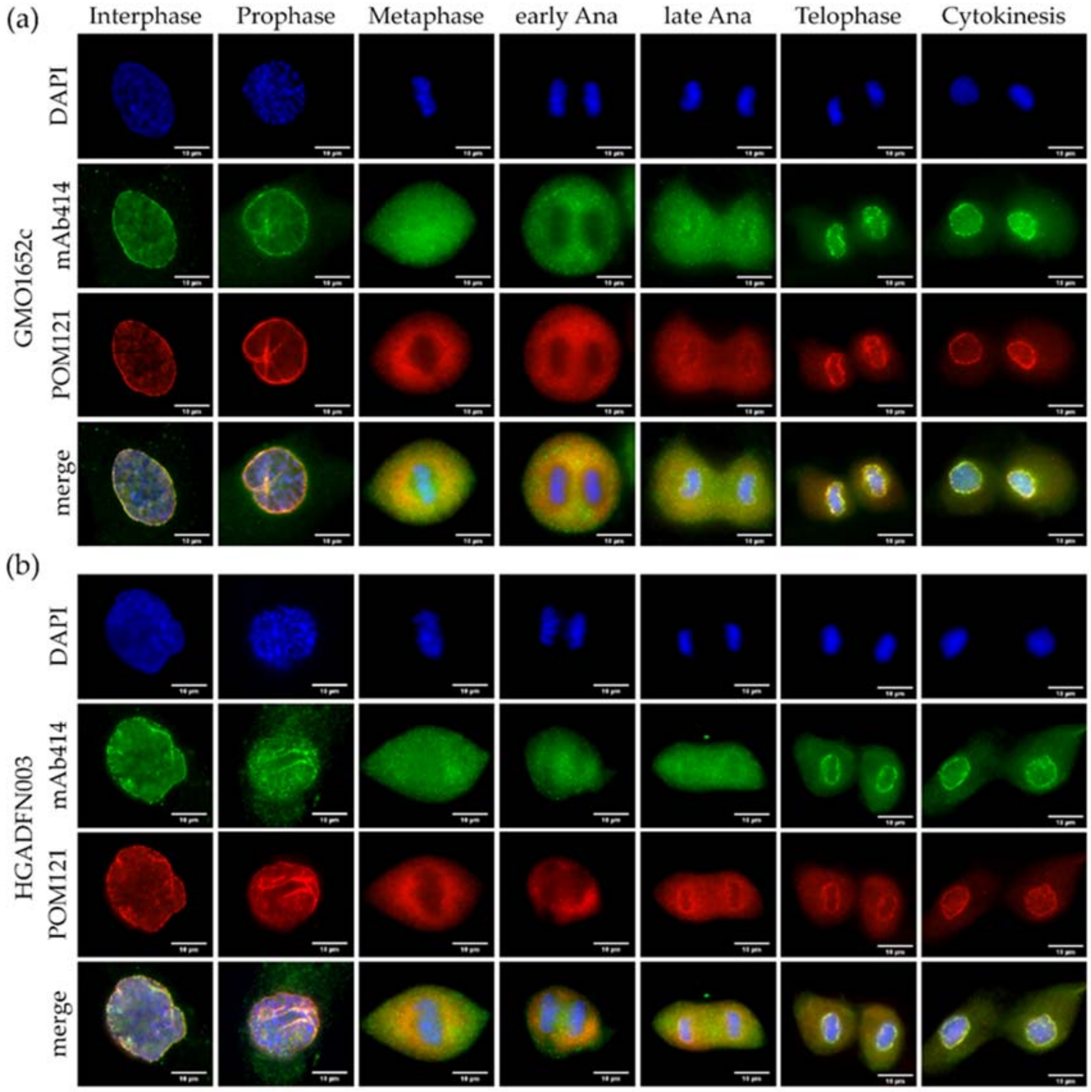


**Figure S4:** Unlike SUN1, POM121 did not form aggregates in HGPS. Immunocytochemistry of (**a**) control (GMO1652c) and (**b**) HGPS (HGADFN003) fibroblasts using mAb414 and $\alpha$-POM121 antibody, counterstained with DAPI. POM121 clusters in invaginations of interphase HGSP cells. Neither the localization nor recruitment of mAb414 and POM121 are affected in mitotic HGPS cells when compared to control. n ≥3.

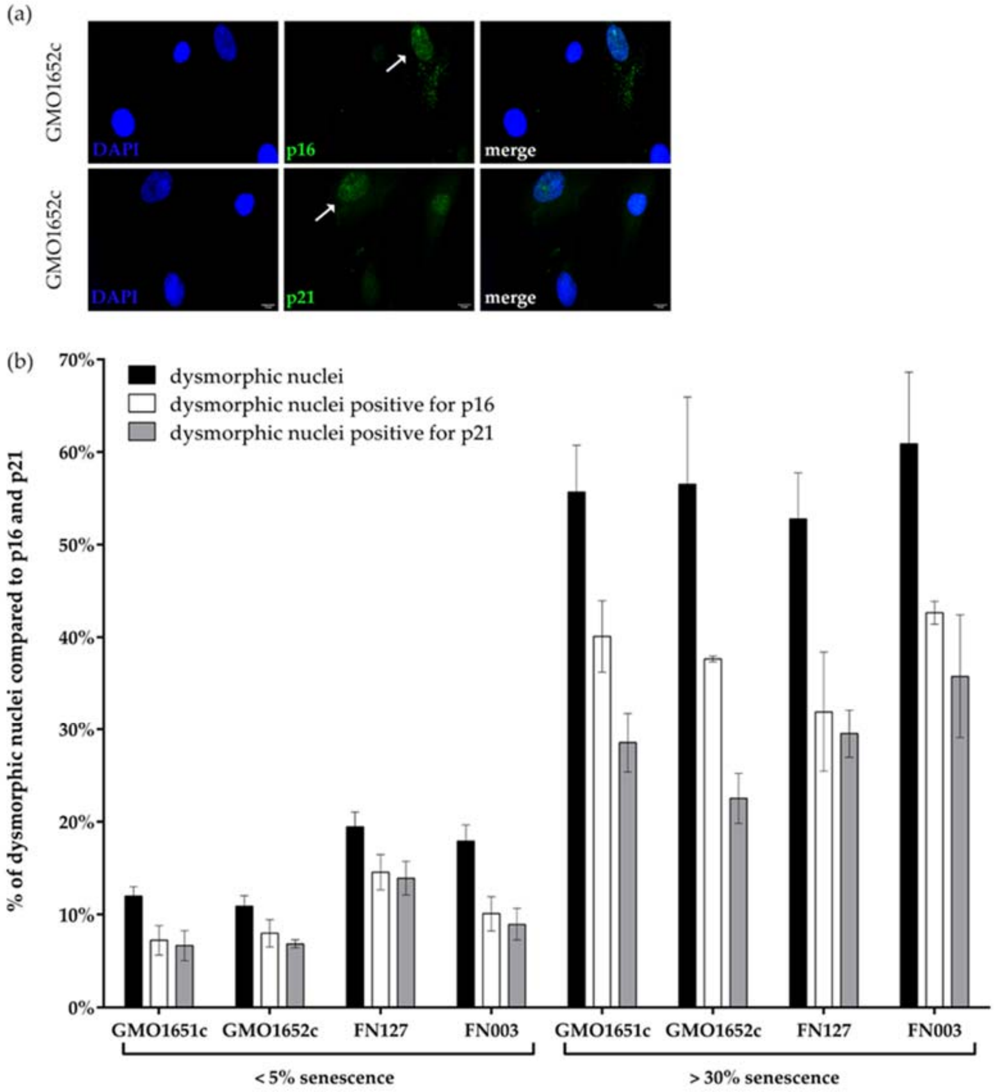


**Figure S5:** Evaluation of senescence markers. (**a**) Representative images of GMO1652c stained with $\alpha$-p1, and $\alpha$-p21, counterstained with DAPI. Arrow points out dysmorphic nuclei with elevated p16 or p21. (**b**) The number of dysmorphic nuclei with elevated p16 orp21 in < 5% and > 30% control and HGPS cells, determined by counting dysmorphic nuclei positive for high p16 or p21, counterstained with DAPI. Before immunocytochemistry, the replicative senescence of cells was determined by SA-β-galactosidase assay. At 5% senescence, HGPS cells had a passage number of ≤ P18, control cells were ≤ P21. At 30% senescence, control was ≥ P25, HGPS was ≥ P21. Values are presented as mean ± SD (n ≥3). SA-β-galactosidase, Senescence-associated beta-galactosidase.

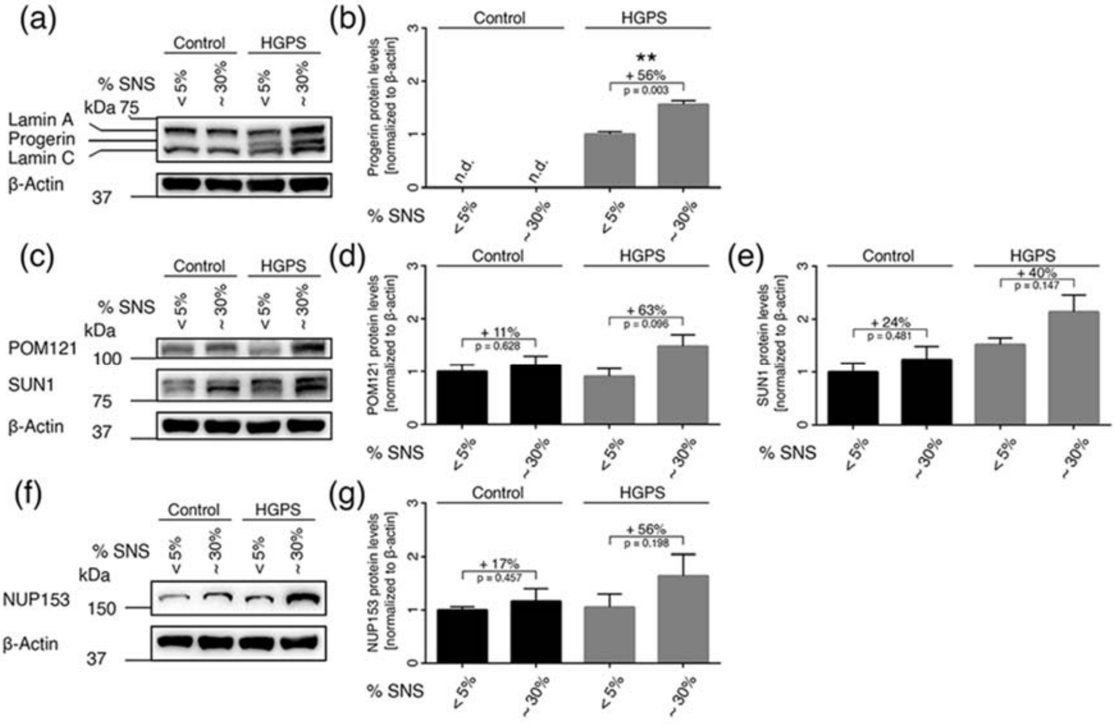


**Figure S6:** Evaluation of NUP153, POM121, SUN1 and Lamin A/C protein levels in control and HGPS. (**a,c,f**) Representative western blot of control (GMO1651c (SNS <5% at passage 15 and SNS ~ 30% at passage 23) and HGPS (HGADFN127 (SNS 5% at passage 12 and SNS ~30% at passage 18) fibroblasts from cultures at the indicated percentages of senescence (SNS). Antibodies directed against NUP153, POM121, SUN1, LA/C and β-actin were used. Graphs show the mean ± SD. Comparisons were performed by two-tailed t test (* $p < 0.05$, ** $p < 0.01$, *** $p < 001$, $n > 3$)

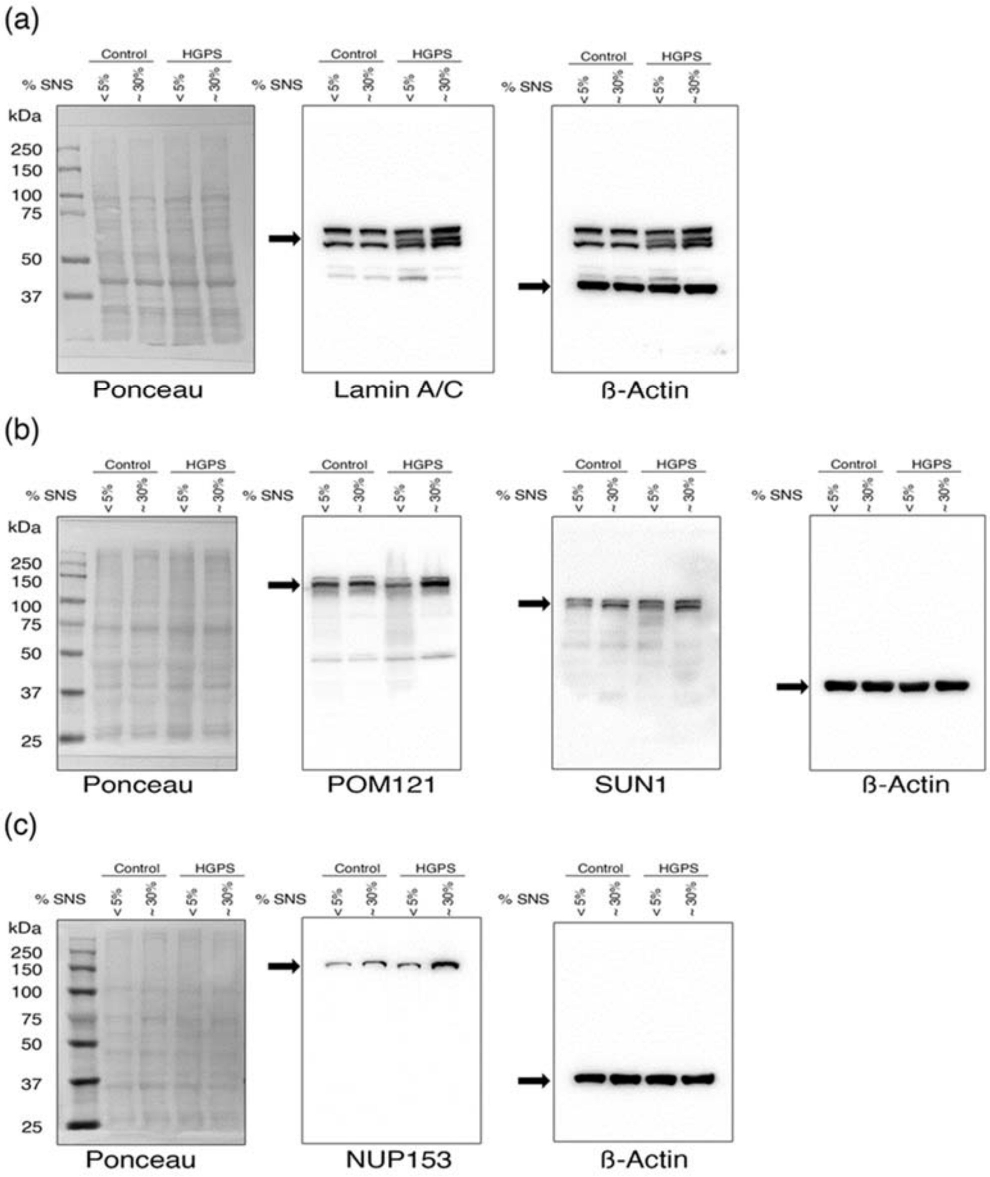


**Figure S7:** Full-length scan of western blots from Figure S6. (**a**) Ponceau and blots of panel Figure S6 a. (**b**) Ponceau and blots of panel Figure S6 c. (**c**) Ponceau and blots of panel Figure S6 f.

Table S1. Number of mitotic cells analyzed.

| SUN1/ mAb414 | Prophase | Metaphase | Anaphase | Telophase | Cytokinesis | Number Experiments |
|---|---|---|---|---|---|---|
| 1651c | 10 | 20 | 26 | 27 | 45 | 11 |
| 1652c | 16 | 30 | 19 | 22 | 59 | 11 |
| P003 | 9 | 12 | 20 | 25 | 34 | 10 |
| P127 | 19 | 26 | 39 | 26 | 36 | 11 |
| **ELYS/ LA** | **Prophase** | **Metaphase** | **Anaphase** | **Telophase** | **Cytokinesis** | **Number Experiments** |
| 1651c | 8 | 31 | 24 | 14 | 31 | 4 |
| 1652c | 9 | 17 | 15 | 10 | 24 | 5 |
| P003 | 8 | 22 | 15 | 22 | 36 | 6 |
| P127 | 7 | 43 | 47 | 23 | 22 | 6 |
| **POM121/ mAb414** | **Prophase** | **Metaphase** | **Anaphase** | **Telophase** | **Cytokinesis** | **Number Experiments** |
| 1651c | 9 | 25 | 60 | 35 | 53 | 10 |
| 1652c | 18 | 16 | 13 | 20 | 31 | 6 |
| P003 | 13 | 25 | 42 | 25 | 31 | 10 |
| P127 | 12 | 22 | 46 | 29 | 56 | 9 |
| **NUP153/ NUP107** | **Prophase** | **Metaphase** | **Anaphase** | **Telophase** | **Cytokinesis** | **Number Experiments** |
| 1651c | 11 | 39 | 31 | 32 | 33 | 5 |
| 1652c | 13 | 20 | 15 | 15 | 29 | 6 |
| P003 | 16 | 19 | 26 | 21 | 23 | 8 |
| P127 | 7 | 16 | 25 | 22 | 48 | 5 |

Table S2. Frequency of mislocalized la, sun1 and npc proteins in mitotic cells.

| **SUN1 localization in HGPS %** | **Metaphase** | **Anaphase** | **Telophase** | **Cytokinesis** |
|---|---|---|---|---|
| no distribution changes | 7.69 ± 7.69 | 16.54 ± 6.54 | 21.8 ± 10.23 | 21.24 ± 6.54 |
| altered distribution | 92.3 ± 7.69 | 83.46 ± 6.54 | 78.20 ± 10.23 | 78.76 ± 6.54 |
| **SUN1 localization in control %** | **Metaphase** | **Anaphase** | **Telophase** | **Cytokinesis** |
| no distribution changes | 79.17 ± 5.83 | 90.89 ± 1.42 | 80.39 ± 12.2 | 83.49 ± 1.26 |
| altered distribution | 20.8 ± 5.83 | 9.11 ± 1.42 | 19.61 ± 12.21 | 16.51 ± 1.26 |
| **Lamin A localization in HGPS %** | **Metaphase** | **Anaphase** | **Telophase** | **Cytokinesis** |
| no distribution changes | 16.28 ± 16.28 | 33.40 ± 13.40 | 30.93 ± 8.20 | 26.14 ± 11.36 |
| altered distribution | 83.72 ± 16.28 | 66.60 ± 13.40 | 69.07 ± 8.20 | 73.86 ± 11.36 |
| **Lamin A localization in control %** | **Metaphase** | **Anaphase** | **Telophase** | **Cytokinesis** |
| no distribution changes | 89.00 ± 5.12 | 89.27 ± 2.50 | 87,86 ± 2,14 | 95.77 ± 4.17 |
| altered distribution | 11.00 ± 5.12 | 10.83 ± 2.50 | 12,14 ± 2,14 | 4.17 ± 4.17 |
| **POM121 localization in HGPS versus control %** | **Metaphase** | **Anaphase** | **Telophase** | **Cytokinesis** |
| no distribution changes | 100.0 ± 0.0 | 100.0 ± 0.0 | 100,0 ± 0,0 | 100.0 ± 0.0 |
| altered distribution | 0.0 ± 0.0 | 0.0 ± 0.0 | 0,0 ± 0,0 | 0.0 ± 0.0 |
| **ELYS localization in HGPS versus control %** | **Metaphase** | **Anaphase** | **Telophase** | **Cytokinesis** |
| no distribution changes | 100.0 ± 0.0 | 100.0 ± 0.0 | 100,0 ± 0,0 | 100.0 ± 0.0 |
| altered distribution | 0.0 ± 0.0 | 0.0 ± 0.0 | 0,0 ± 0,0 | 0.0 ± 0.0 |
| **NUP107 localization in HGPS versus control %** | **Metaphase** | **Anaphase** | **Telophase** | **Cytokinesis** |
| no distribution changes | 100.0 ± 0.0 | 100.0 ± 0.0 | 100,0 ± 0,0 | 100.0 ± 0.0 |
| altered distribution | 0.0 ± 0.0 | 0.0 ± 0.0 | 0,0 ± 0,0 | 0.0 ± 0.0 |
| **NUP153 localization in HGPS versus control %** | **Metaphase** | **Anaphase** | **Telophase** | **Cytokinesis** |
| no distribution changes | 100.0 ± 0.0 | 100.0 ± 0.0 | 100,0 ± 0,0 | 100.0 ± 0.0 |
| altered distribution | 0.0 ± 0.0 | 0.0 ± 0.0 | 0,0 ± 0,0 | 0.0 ± 0.0 |
